# Examining the changes in women's lives after the hysterectomy operation: Experiences of women from Turkey

**DOI:** 10.1007/s00737-024-01419-3

**Published:** 2024-01-06

**Authors:** Ayşenur Turan, Hilal Başak Karabayır, İffet Güler Kaya

**Affiliations:** Department of Midwifery, Faculty of Health Sciences, Istanbul Medipol University, Istanbul, Turkey

**Keywords:** Body image, Femininity, Hysterectomy, Psychology of women after the operation, Self-perception

## Abstract

**Purpose:**

The aim of the study is to examine what kind of changes the operation has brought about in the social and psychological life of women.

**Methods:**

The study was a hermeneutic-phenomenological research conducted using qualitative research methods. It took place between May and July 2023 at a university hospital located in Istanbul, Turkey. Following ethical approval, the study included a total of 24 women who had undergone a 'total abdominal hysterectomy, bilateral salpingo-oophorectomy.' These participants were selected using a combination of maximum diversity sampling and random sampling methods.

Data collection was carried out using a semi-structured interview form. The interviews were conducted using face-to-face interview techniques and in-depth interview methods. Qualitative data analysis involved using the coding paradigm of Grounded Theory and Straus and Corbin's coding framework. For the analysis of quantitative data, SPSS version 28.0 was employed, while qualitative data were analyzed using MaxQDA Analytics Pro 2022.

**Results:**

The study found that the participants had a mean age of 47 ± 7.53, and the majority, 66.7%, had not reached menopause before the operation. The qualitative analysis of the semi-structured interviews revealed five main themes, which were identified as follows: 'hysterectomy experiences', 'the impact of hysterectomy on sexual life', 'the significance attributed to the uterus', 'the significance attributed to femininity', and 'self-efficacy after hysterectomy'.

**Conclusions:**

The study revealed that women experienced changes in self-perception, body image, a sense of organ loss, and developed negative attitudes towards menopause following a hysterectomy. Additionally, there was a noticeable connection between the meanings attributed to the uterus and femininity, which was often influenced by cultural and social factors. In light of these findings, it is recommended that women receive counseling from healthcare professionals before undergoing a hysterectomy. This guidance can help women better understand and cope with the physical, emotional, and cultural aspects of the procedure.

## Introduction

Hysterectomy, the most prevalent gynecological procedure globally, involves the removal of the uterus for various medical reasons (Lee et al. 2019). This surgery can be carried out through different methods, including abdominal, vaginal, and laparoscopic approaches. The total removal of the uterus, cervix, fallopian tubes, and ovaries via an abdominal method is referred to as 'total abdominal hysterectomy and bilateral salpingo-oophorectomy' (Huang et al. 2020). The specific technique used for hysterectomy may vary depending on the surgical indication, patient characteristics, surgeon expertise, and individual preferences. Indications for hysterectomy encompass irregular bleeding, menstrual disorders, fibroids, pelvic pain, uterine prolapse, as well as other reasons like endometrial diseases, reproductive system cancers, and localized genital endometriosis within the myometrium (Huang et al. 2020; Ghomi et al. 2021). The incidence rates of hysterectomy vary worldwide, with developed countries reporting higher rates. The likelihood of undergoing hysterectomy increases with age. For instance, in the United States, the rate of hysterectomy among women aged 20 to 29 is less than 2%, while more than 4 out of 10 women between the ages of 70 and 79 have undergone hysterectomy (Adam et al. 2022; Lycke et al. 2021).

Surgical interventions have a profound impact on an individual's body integrity, self-perception, quality of life, and psychosocial well-being. Gynecological procedures such as hysterectomies add additional stressors, affecting women's reproductive capacity and sexual functions (Hsieh et al. 2021). Furthermore, undergoing such surgery is a risk factor for anxiety, social dysfunction, and depression, as women may become infertile post-procedure (Campbell et al. 2017; Skorupska et al. 2021).

The uterus holds multiple symbolic meanings for women, including being a sexual organ, a fertility organ, a secretory organ, a symbol of youth, motherhood, attractiveness, and a source of power. Cultural factors play a significant role in shaping these perceptions (Erdogan et al. 2020; Hsieh et al. 2021). Consequently, a hysterectomy is not only the loss of a valued organ but also a crucial procedure that impacts women physically, emotionally, and sexually (Till et al. 2022).

A hysterectomy is a surgical procedure that can have emotional and psychological consequences for women. In various cultural contexts, the uterus is often seen as a symbol of femininity and fertility. Consequently, women's attitudes and perceptions concerning this procedure can vary significantly from one culture to another, as highlighted by the research carried out by Hsieh et al. (2021) and Skorupska et al. (2021). While there are studies in the literature examining emotional changes after hysterectomy, the number of studies conducted in the specific country of research is limited (Pinar et al. 2012; Erdogan et al. 2020). Considering that cultural factors can influence the meanings associated with the uterus (Erdogan et al. 2020; Hsieh et al. 2021), this study aims to explore the social and physiological changes that Turkish women may undergo after a hysterectomy.

The aim of the study is to examine what kind of changes the operation has brought about in the social and psychological life of women.

## Material and Methods

### Study design and setting

This research was conducted as a hermeneutic-phenomenological study, one of the qualitative research methods, in a University Hospital in Istanbul/Turkey between May and July 2023 to examine the feelings and thoughts of women who had hysterectomy operations.

Hermeneutics means that it should target discursive language and sensitive interpretive tools that make it possible and understandable to reflect on the experience and explain or describe phenomenological analysis (Van Manen and Van Manen 2021). Hermeneutic phenomenology is a method of reflecting soberly on the basic structures of the lived experience of human existence. It focuses on the phenomena-lived experiences of individuals, how they make sense of the process, and how they transform their experiences into consciousness. In this approach, the phenomenon is analysed, and the factors that make up the experience are tried to be determined (Korstjens & Moser 2017; Errasti-Ibarrondo et al. 2018; Van Manen & Van Manen 2021).

The choice of employing the harmonic phenomenology method in this research, as opposed to the literal definitions method, is aimed at illuminating how women derive meaning from their experiences of hysterectomy and the subsequent physical and social changes.

### Study participants

In qualitative research, data saturation is reached when no new analytical information emerges, and the study provides maximum information about the phenomenon. The usually small sample size in qualitative research depends on the information richness of the data, the diversity of participants, the breadth of the research question and phenomenon, the method of data collection (e.g., individual or group interviews), and the type of sampling strategy. The most important criterion for determining the sample is sufficient in-depth data showing the patterns, categories, and diversity of the phenomenon under study (Korstjens and Moser 2018). In this study, data saturation was reached with 24 participants. The research was conducted with 24 participants.

Following ethical approval, a total of 24 women who had undergone a 'total abdominal hysterectomy, bilateral salpingo-oophorectomy' were selected for the study through a combination of maximum diversity sampling and random sampling methods.

The maximum variation sampling method is employed with the objective of creating a relatively small sample that maximally represents the diversity of individuals who could be relevant to the issue under investigation (Palinkas et al. 2015; Korstjens and Moser 2018). The rationale behind utilizing the maximum diversity sampling method in this research is to ensure the inclusion of participants who can collectively represent all segments of the society where the study is conducted. This approach aims to yield results that are reflective of the broader society. In the present study, factors such as education, income, and place of residence were taken into account to ensure maximum diversity in the sample. No restrictions were imposed concerning age, education, and employment status to enhance the richness of the study's data. The study did not include women with a previous history of uterine cancer.

### Data Collection

Data were collected through a ‘semi-structured interview form’ consisting of 17 questions. The semi-structured interview questionnaire was developed based on previous research on the topic (Erdogan et al. 2020; Hsieh et al. 2021). Initially, the questionnaire comprised a total of 20 questions. These questions were submitted to two experienced qualitative research researchers who were not involved in the current study for internal validity assessment. Two of the questions were eliminated from the questionnaire because they were unclear, and one was redundant with another question. A pilot study was then conducted with three women using the remaining 17 questions. The feedback received indicated that no further adjustments were necessary, as the questions were found to be clear and easily answerable. The semi-structured interview questionnaire consisted of 12 questions, encompassing socio-demographic and obstetric characteristics of the participants (age, educational status, employment status, income level, marital status, place of residence, pre-operative menopause status, number of gravida-parity-abortus-living children, number of abortions). In addition, there were 5 open-ended questions designed with reference to the existing literature (Erdogan et al. 2020; Hsieh et al. 2021) to provide guidance to the researcher. Here are some examples of the questions:

- What significance does this surgery hold for you?
- What positive aspects do you associate with this surgery?
- What negative aspects do you associate with this surgery?
- How do you perceive the importance of the uterus?
- How do you define femininity?

These questions were used to guide the interviews and gather comprehensive information from the participants. Individual in-depth interviews were collected on the second post-operative day (post-operative 20th hour) by face-to-face interview method through a ‘semi-structured interview form’. The interviews were conducted in the women's individual rooms prior to their discharge, when they were free from pain and discomfort. Prior to the interviews, women were asked about their comfort preferences, and they commonly expressed feeling most at ease when they were in their own clothing, without the angiocath, and without anyone else in the room. When asked about situations that might cause discomfort, participants mentioned that unexpected intrusions into the room made them uncomfortable. Consequently, a "do not disturb" sign was placed on the door of the participants' rooms scheduled for interviews, and the interviews took place after the removal of the angiocath and when the participants were dressed in their own clothes.

All interviews were carried out by the corresponding author (A.T), who holds a PhD degree, is female, and possesses prior experience in qualitative research. The author visited the hospital solely for the purpose of conducting this study to prevent any pre-existing bias in her relationship with the participants. The study's objectives were thoroughly explained to the participants. Interviews were conducted in the local language, and both oral and written consent for audio recording were obtained at the outset of each interview. On average, the interviews lasted approximately 15 min. In addition to the open-ended questions, the interviewer asked more detailed follow-up questions based on the women's responses during the interview. Field notes were taken as needed during the interviews, and no repeat interviews were conducted.

### Trustworthiness

Trustworthiness refers to the quality, authenticity, and truthfulness of findings of qualitative research, and it relates to the degree of trust, or confidence, readers have in results (Cypress 2017; Korstjens and Moser 2018). Studies have reported that researchers can use Lincoln and Guba’s (1985) criteria, including credibility, reliability, confirmability, and transferability, to ensure the trustworthiness of qualitative research (Cypress 2017; Korstjens and Moser 2017, 2018; Zhan et al. 2023). The 4 major traditional criteria are summarised into 4 questions about truth value, applicability, consistency, and neutrality. From these, they proposed 4 analogous terms within the naturalistic paradigm to replace the rationalistic terms: credibility, transferability, dependability, and confirmability (Lincoln and Guba 1985). In this study, feedback was obtained from each participant to increase internal reliability. No changes to the findings were requested following this feedback process. In coding and analysing the data, support was received from an independent external expert competent in qualitative research. The Cohen κ for the intercoder reliability was 0.92 (Bakeman 2022). Inclusion and exclusion criteria were determined to increase external reliability, and purposive sampling was used. The researcher used the triangulation method in order to increase reliability and objectivity. We intentionally applied a theory-generating design in which we used no specific theory to create the interview guide, purposive sampling techniques to target specific subgroups of women, and standardisation of interviews across all participants to enhance the validity of findings and to ensure that the results represented the true experiences of participants. We enhanced consistency and adherence to established standards for qualitative analysis by holding regular meetings (Cypress 2017). The authors engaged in rigorous discussions and reflection over several months, soliciting feedback from an experienced qualitative researcher not involved in the study to ensure that the findings were representative of the participants’ narratives and experiences and not tainted by the authors’ experiences and biases (Cypress 2017).

### Data analysis

To evaluate quantitative data, mean values, minimum and maximum values, and standard deviation were used. For the analysis of qualitative data, the content analysis method was applied. After each interview, the responsible researcher (A.T) transcribed the audio recordings using the online platform (https://voiser.net), which transcribes audio into text on the computer. The transcriptions were independently reviewed by a co-author (B.K). Subsequently, co-author (B.K) developed the initial coding structure, which was then presented to the other two researchers involved in the study. Qualitative data analysis followed the coding paradigms of Grounded Theory and Corbin and Strauss’ coding paradigm (Corbin & Strauss 1990). This involved creating open codes and categorization (axial coding), from which themes (selective coding) were derived. These themes were generated based on the data collected.

To ensure the rigor and reliability of the analysis, a consensus was reached among the researchers in the development of themes following the existing literature. After the initial themes were established, input was sought from five experts, and the themes were finalized based on their recommendations. This process aimed to enhance the validity and robustness of the qualitative data analysis.

In the presentation of the data, participant names were not used, and each participant was identified by a corresponding number (P1, P2, … P24). For the analysis of quantitative data, the Statistical Package for the Social Sciences (IBM SPSS Statistics for Macintosh, Version 28.0., Armonk, NY: IBM Corp) was utilized. Qualitative data were analyzed using MaxQDA Analytics Pro 2022, a qualitative data analysis software developed by VERBI Software in Berlin, Germany.

### Ethical consideration

Before commencing data collection, the study received approval from the local ethics committee (Decision Date: 08.05.2023; Decision Number: 61) and obtained institutional permission from the organization where the research was conducted. The study was conducted in accordance with the Declaration of Helsinki and adhered to the ethical standards of the country of origin. Furthermore, all participants provided written and oral informed consent before participating in the study.

## Results

### Characteristic of the study participants

The study included 24 women who underwent total abdominal hysterectomy and bilateral salpingo-oophorectomy. The mean age of the participants was 47 ± 7.53 years. It was found that 79.2% (n = 19) of the participants were married, 65% (n = 13) had less income than expenses, 41.7% (n = 10) were primary school graduates, 58.3% (n = 14) were not working, and 54.2% (n = 13) had no chronic disease. It was determined that 50% (n = 12) of the participants had 3 and 5 previous pregnancies and 75% (n = 18) had 0 and 3 living children (Table 1).

**Table 1:** Characteristic of the participants (N = 24)

Characteristics	n	%
Marital status		
Married Single	19	79.2
	5	20.8
Educational status		
Literate Primary school Secondary school High school University	3	12.5
	10	41.7
	4	16.7
	6	25.0
	1	4.2
Employment status		
Not working	10	41.7
Working	14	58.3
Economic Level		
Income less than expenditure Income equals expenditure Income more than expenditure	9	37.5
	11	45.8
	4	16.7
Longest lived in		
Province District Town Village	16	66.7
	6	25
	1	4.2
	1	4.2
Chronic Disease		
No Yes	13	54.2
	11	45.8
Gravida		
0–2	7	29.2
3–5	12	50
6–8	5	20.8
Number of Children Alive		
0–3	18	75.0
4 and above	6	25.0
Number of Spontaneous Abortions		
0	19	79.2
1 and above	5	20.9
Preoperative menopausal status		
No	16	66.7
Yes	8	33.3
	**Mean/ SD**	**Min–Max**
Age	47 ± 7.53	30–62

The data obtained from the interviews were collected under 5 main themes (hysterectomy experiences, the effect of hysterectomy on sexual life, meaning attributed to the uterus, the meaning attributed to femininity, self-efficacy after hysterectomy). Examples of themes and sub-themes are presented below.

### Hysterectomy Experiences

**Fig. 1:**
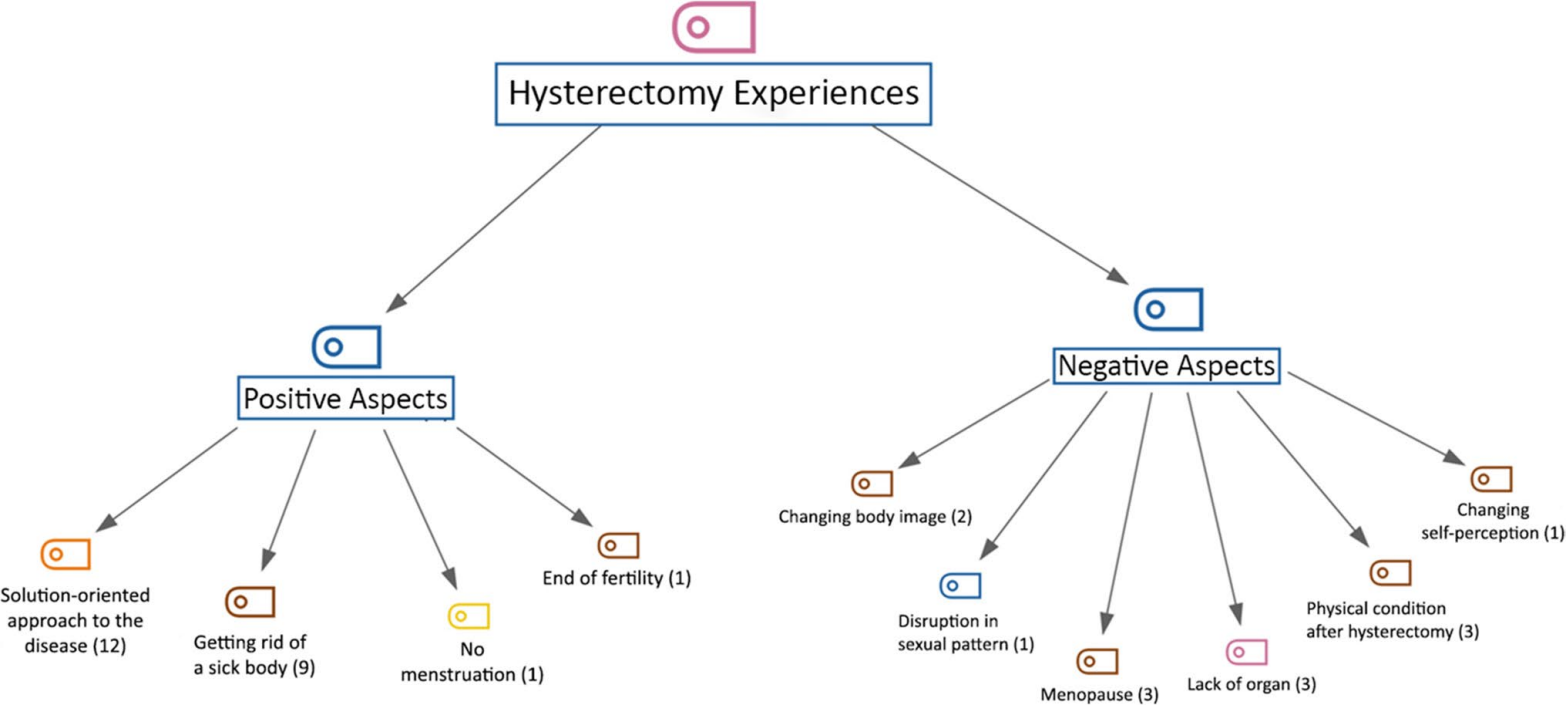
Hierarchical Code Subcode Model of Hysterectomy Experiences

The hierarchical code-subcode model of the first theme of the study, women’s ‘hysterectomy experiences’, is shown in Fig. 1. Women’s hysterectomy experiences were coded as ‘positive aspects’ (n = 23) and ‘negative aspects’ (n = 13). In the ‘positive aspects’ code of the theme of women’s hysterectomy experiences, the concepts of ‘solution-oriented approach to the disease’, ‘getting rid of a sick body’, ‘not seeing menstruation’ and ‘ending fertility’ were coded as sub-codes. In the ‘negative aspects’ code of the theme of women’s ‘hysterectomy experiences’, the concepts of’organ deficiency’, ‘physical condition after hysterectomy’, ‘menopause’, ‘changing body image’, ‘changing self-perception’, ‘disruption in sexual pattern’ were coded as sub-codes (Fig. 1).

In the sub-code ‘solution-oriented approach to the disease’ (n = 12) (Fig. 1), the statements of the participants coded P3, P16, P17, and P23 on the subject are as follows:

P3 (age: 40; gravida: 5):* I had a lot of menstrual bleeding before coming to the hospital, I had more than 10 days of bleeding, and the doctor at the hospital said that cancer cells had formed and that I had to have this surgery before I passed the stage, I was very scared, but I had to accept the surgery so that I could survive.*

P16 (age: 42; gravida: 3):* During my menstrual days, I would bleed all over the bed sheets and sofa, making my life difficult. I was suffering from anaemia, and although the surgery made me uneasy, I think it helped me a lot.*

P17 (age: 53; gravida: 4):* I accepted because I would get rid of cancer. If there had been a cure, I would not have had surgery.*

P23 (age: 43; gravida: 1): *It is a necessary surgery for my health. I had to have it, and I did.*

In the sub-code ‘getting rid of a sick body’ (n = 9) (Fig. 1), the statements of participants coded P4 and P14 on this topic are as follows:

P4 (age: 62; gravida: 5): *Since my uterus was removed entirely, the discomfort caused by sagging has disappeared.*

P14 (age: 60; gravida: 6): *Compared to the preoperative period, my pain has decreased, I feel better, and I feel more comfortable.*

The statements of the participant coded P2 in the sub-code of ‘ending of fertility’ (n = 1) (Fig. 1) are as follows:

P2 (age: 43; gravida: 4): *I got married at 18, and I have had two normal and two caesarean births so far. I am happy that I will not get pregnant anymore.*

The statements of the participant coded P1 in the sub-code ‘not menstruating’ (n = 1) (Fig. 1) are as follows:

P1 (age: 35; gravida: 0): *I will not have menstrual pain every month, I will not wear pads, so I am happy.*

The statements of the participants coded P1, P16 and P18 in the sub-code ‘organ deficiency’ (n = 3) (Fig. 1) are as follows:

P1 (age: 35; gravida: 0):* Since I do not want children, there is no harm for me in having this surgery, but it is still a negative side for me that I no longer have the reproductive organ that should normally be in the female body.*

P16 (age: 42; gravida: 3): *It feels strange for me to have my uterus removed. It is a part of my body, and even though I don’t want to have children, not having a uterus makes me feel incomplete.*

P18 (age: 47; gravida: 3): *I feel like I am missing an organ and uncomfortable because they cut my uterus.*

In the sub-code ‘physical condition after hysterectomy’ (n = 3) (Fig. 1), the statements of the participants coded P4 and P6 on the subject are as follows.

P4 (age: 62; gravida: 5): *My operation was open, and my recovery took a long time.*

P6 (age: 43; gravida: 4): *I suffered a lot of gas pains, my surgery took a long time, I could not eat, and I suffered a lot of nausea.*

In the sub-code ‘menopause’ (n = 3) (Fig. 1), the statements of the participants coded P13 and P15 are as follows:

P13 (age: 55; gravida: 6): *The thought of going into menopause caused me a lot of distress; I get a fever when I think about it.*

P15 (age: 43; gravida: 4): *I am worried that I will reach menopause after the operation.*

In the ‘body image’ (n = 2) sub-code (Fig. 1), the statements of the participants coded P13 and P21 on the subject are as follows:

P13 (age: 55; gravida: 6): *Now that I have reached menopause, I feel like this; I feel like a man.*

P21 (age: 50; gravida: 3): *With the absence of breasts, my uterus will now be removed. It makes me feel like I have lost my femininity, and I feel very sad.*

The statements of the participant coded P20 in the sub-code ‘changing self-perception’ (n = 1) (Fig. 1) are as follows:

P20 (age: 45; gravida: 3): *I have never been a gorgeous woman, but after this surgery, I think there will be changes in my body because I will reach menopause, and this worries me.*

The statements of the participant coded P3 in the sub-code ‘disruption in sexual pattern’ (n = 1) (Fig. 1) are as follows:

P3 (age: 40; gravida: 5): *I am worried that it will negatively affect my sexual life.*

### The Effect of Hysterectomy on Sexual Life

**Fig. 2:**
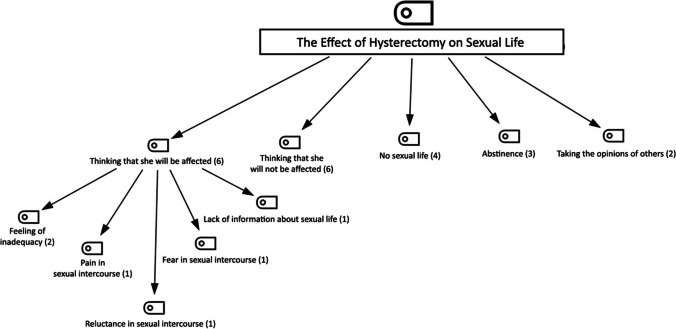
Hierarchical Code Sub-Code Model of the Effect of Surgery on Sexual Life

The hierarchical code-subcode model for another study theme, ‘the effect of hysterectomy on sexual life’, is shown in Fig. 2. The codes of ‘hysterectomy affecting sexual life’ are ‘thinking that she will be affected’, ‘thinking that she will not be affected’, ‘no sexual life’, ‘abstinence’, ‘feeling of inadequacy’, and ‘taking the opinions of others’. Subcodes were created only in the code ‘thinking that she will be affected’, and these subcodes were ‘feeling of inadequacy’, ‘pain in sexual intercourse’, ‘reluctance in sexual intercourse’, ‘fear in sexual intercourse’, and ‘lack of information about sexual life’ (Fig. 2).

The statement of the participant coded P21 in the code ‘feeling of inadequacy’ (n = 2) (Fig. 2), which is a sub-code of ‘thinking that she will be affected’ (n = 6), is as follows:

P21 (age: 50; gravida: 3): *Sexually, I have concerns about my husband. I can’t help but wonder if he will feel a difference. I have a feeling that it will affect him negatively.*

The statement of the participant coded P19 in the sub-code ‘reluctance in sexual intercourse’ (n = 1) (Fig. 2) is as follows:

P19 (age: 30; gravida: 2):* I think it will have a negative effect for a while, but it will not be a problem later. I can return to the old days; there may be sexual reluctance.*

The statements of the participants coded P3 and P9 in the sub-code ‘fear in sexual intercourse’ (n = 1) (Fig. 2) are as follows:

P3 (age: 40; gravida: 5): *I think it won’t be nice; I mean, I think it will not be like the relationship before my uterus was removed for me and my husband.*

P9 (age: 49; gravida: 2): *I will be passive in terms of femininity. I feel fear, anxiety, and inadequacy.*

The statement of the participant coded P22 in the sub-code ‘pain in sexual intercourse’ (n = 1) (Fig. 2) is as follows:

P22 (age: 40; gravida: 2): *I think I will have painful sexual intercourse.*

The statements of the participants coded P3 and P12 in the sub-code ‘lack of information about sexual life’ (n = 1) (Fig. 2) are as follows:

P3 (age: 40; gravida: 5): *I lost an organ after the operation. I have no information about how this will affect my life and my relationship, but I heard from the people around me that it has no negative effects; they said that when one of the kidneys is removed, it will be the same as when we continue everyday life, and I am so worried.*

P12 (age: 50; gravida: 8): *I do not know this yet, but I think it will affect me; it puts a question mark in my mind.*

Another code of the theme of the effect of the surgery on sexual life is the code of thinking that she will not be affected (n = 6) (Fig. 2). The statements of the participants coded P16, P20 and P24 are as follows:

P16 (age: 42; gravida: 3): *Actually, I searched for this issue on the internet, and this surgery does not have much effect on sexual relations. Since I am already in my 40s, I am not looking for sexual relations much. After a certain age, such desires do not remain much. It will not affect me much.*

P20 (age: 45; gravida: 3): *If no one had told me that my uterus had been removed after the operation, I would not have known that my uterus had been removed. That’s the truth!*

P24 (age: 50; gravida: 2): *I am a person who does not like sexual activity. I do not think it will be affected by this surgery. I was already having difficulty with sexual relations. Now I can talk to my husband more easily.*

Another code of the theme of hysterectomy affecting sexual life is ‘no sexual life’ (n = 4) (Fig. 2). The statements of the participants coded P1 are as follows:

P1 (age: 35; gravida: 0): *Since I did not have a sexual life before the operation, I think that there will be no change in my sexual life after the operation. I know there will be no change in my satisfaction since there is no change in my external genital organs.*

In another code, ‘abstinence’ (n = 3) (Fig. 2), the statements of the participants coded P6 and P11 are as follows:

P6 (age: 43; gravida: 5): *I don’t think about these things at all; I am struggling with my life right now.*

P11 (age: 43; gravida: 5): *I don’t think about that right now. We will experience it when the time comes.*

In the last code, ‘taking the opinions of others’ (n = 2) (Fig. 2), the statements of the participants coded P18 and P20 are as follows.

P18 (age: 47; gravida: 3): *I asked my friends and neighbours, and I heard that it may have negative effects on pleasure.*

P20 (age: 45; gravida: 3): *I asked a lot before the surgery. I even went and asked older women in parks. I asked people I did not know. I also went to a psychiatrist; obviously, the psychiatrist did not satisfy me. I wanted to learn from those who had experienced it. Everyone said there was no such thing. I don’t think I have accepted that thought since the day I had the operation.*

### Meaning Attributed to the Uterus

**Fig. 3:**
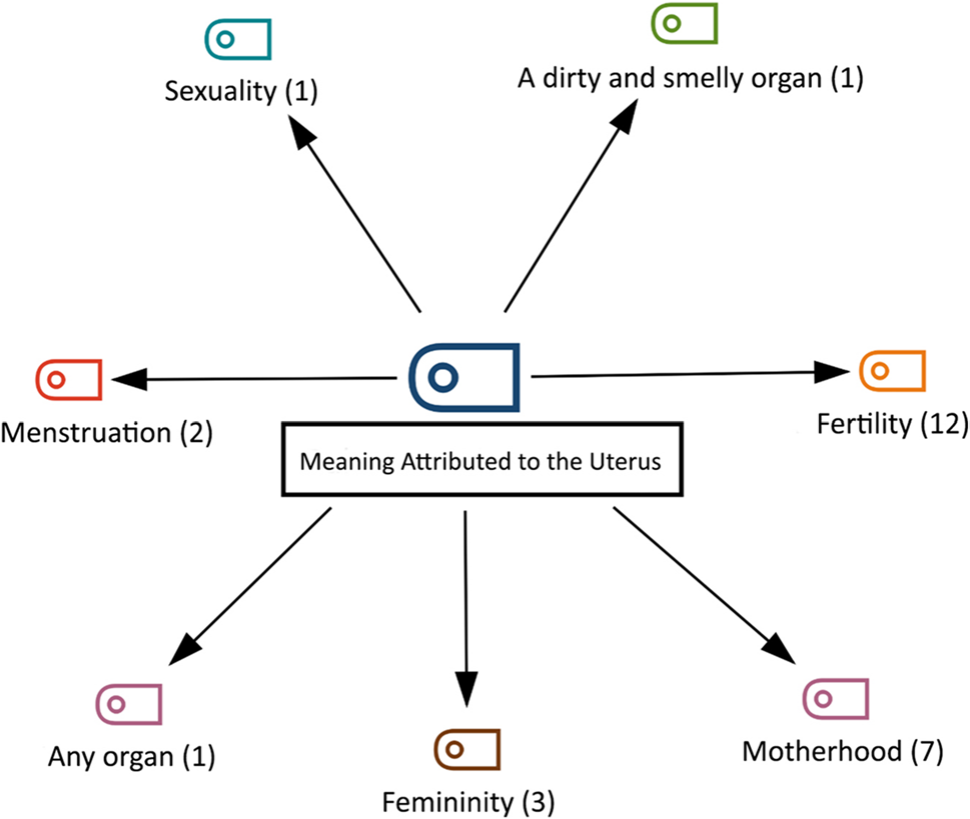
The Hierarchical Code Sub-Code Model of the Theme of the Meaning of Uterus

The hierarchical code-subcode model for the themes related to the meaning attributed to the uterus, which is another theme of the research, is shown in Fig. 3. The codes belonging to the theme ‘meaning attributed to the uterus’ (n = 27) are ‘fertility’, ‘femininity’, ‘motherhood’, ‘menstruation’, ‘sexuality’, ‘a dirty and smelly organ’, ‘any organ’ (Fig. 3).

The most frequently expressed code was fertility (n = 12) (Fig. 3). The statements of the participants coded P1, P14, P18, and P24 are as follows:

P1 (age: 35; gravida: 0): *I see it as a home where a living being can grow.*

P14 (age: 60; gravida: 6): *The uterus is the place where the baby develops and grows.*

P18 (age: 47; gravida: 3): *It is an organ that can make children. It is a perfect organ for a woman and her husband. After this age, I think it does not mean anything; it is an organ that may or may not be.*

P24 (age: 50; gravida: 2): *For me, it means a baby, so I think that if you are not going to have a baby, there is no need for a uterus.*

The statements of the participants coded P12, P21 in the ‘motherhood’ (n = 7) code (Fig. 3) are as follows:

P12 (age: 50; gravida: 8): *The uterus reminds me of a child.*

P21 (age: 50; gravida: 3): *It is the place where my children, my most valuable assets in this life, are formed. It is an extraordinary organ for me.*

In the femininity (n = 3) code (Fig. 3), the statements of the participants coded P4, P10 and P15 are as follows:

P4 (age: 62; gravida: 5): *The most important organ of a woman is the uterus.*

P10 (age: 50; gravida: 5): *It expressed my femininity; I feel like I am incomplete, anyway…*

P15 (age: 43; gravida: 4): *It expresses being a woman.*

In the code ‘menstruation’ (n = 2) (Fig. 3), the statements of the participants coded P7 and P22 are as follows:

P7 (age: 48; gravida: 8): *I don’t know how to say the uterus; I don’t know what it means. When menstruation is regular, the person is more comfortable. Since I will reach menopause, I will experience the effects of menopause, such as fever, etc. The uterus refers to menstruation, with menstruation, dirty blood goes away and clean blood comes.*

P22 (age: 40; gravida: 2): *It purifies the dirty blood in my body*.

The statements of the participant coded P13 in the code ‘a dirty and smelly organ’ (n = 1) (Fig. 3) are as follows:

P13 (age: 55; gravida: 6): *It expresses where the child is. I wish they had removed it after my last child instead of this happening to me. The stench always comes from there.*

In the ‘sexuality’ code (n = 1) (Fig. 3), the statements of the participant coded P3 are as follows:

P3 (age: 40; gravida: 5): *I honestly think that it affects sexual intercourse.*

In the ‘any organ’ code (n = 1) (Fig. 3), the statements of the participant coded P11 are as follows:

P11 (age: 43; gravida: 5): *It doesn’t mean anything (pointing to the quilt on her); it means quilt.*

### The Meaning Attributed to Femininity

**Fig. 4:**
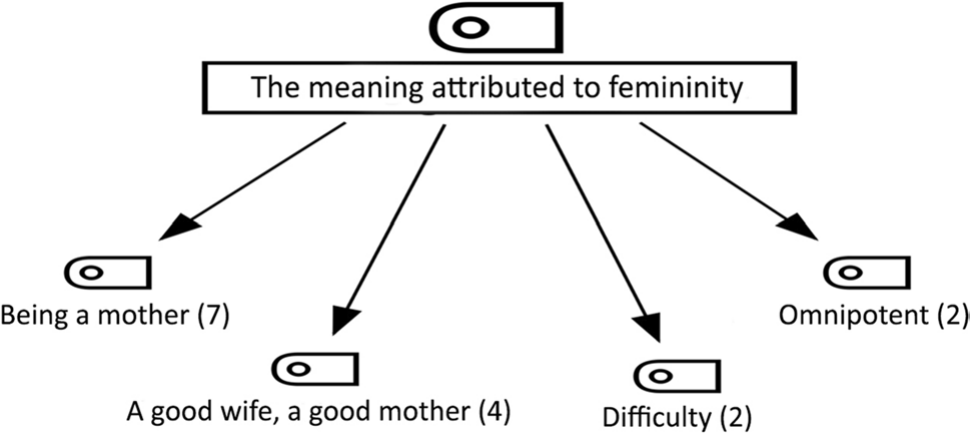
The Hierarchical Code Sub-Code Model of the Theme of the Meaning of Femininity

The hierarchical code sub-codes (n = 15) belonging to the theme of ‘meaning attributed to femininity’, which is another theme of the research, are shown in Fig. 4. The codes belonging to the theme of the meaning attributed to femininity are ‘being a mother’, ‘a good wife, a good mother’, ‘difficulty’, and ‘omnipotent’ (Fig. 4).

The most intensely expressed code of the theme of the meaning attributed to femininity is ‘being a mother’ (n = 7) (Fig. 4). The statements of the participants coded P1, P12, and P22 on the subject are as follows:

P1 (age: 35; gravida: 0): *Being a woman means going beyond the gender roles we are taught. I do not see myself as a machine that only does housework and tidies up. I am aware of my wishes and needs. I think it also means motherhood. I am not the mother of a child but of 2 cats.*

P12 (age: 50; gravida: 8): *Being a mother and having children.*

P22 (age: 40; gravida: 2): *It means being a mother.*

The statements of the participants coded P15 in the ‘good wife, good mother’ code (n = 4) (Fig. 4) are as follows:

P15 (age: 43; gravida: 4): *It means being a good wife and mother who takes care of the household chores and cares for her children and husband.*

In the ‘difficulty’ code (n = 2) (Fig. 4), the statements of the participants coded P14 and P17 are as follows:

P14 (age: 60; gravida: 6): *I think being a woman is not a good thing. There is a workload; there are difficulties, such as childbearing.*

P17 (age: 53; gravida: 4): *Ordeal, being a woman is an ordeal; it never ends.*

In the ‘omnipotent’ code (n = 2) (Fig. 4), the statements of the participants coded P16 are as follows:

P16 (age: 42; gravida: 3): *Being a woman is very tiring because it means spending time on housework, children, and husband. Because you have to be enough for all of them, sometimes I neglect myself. I mean, struggling both for yourself and for your whole family.*

### Self-efficacy After Hysterectomy

**Fig. 5:**
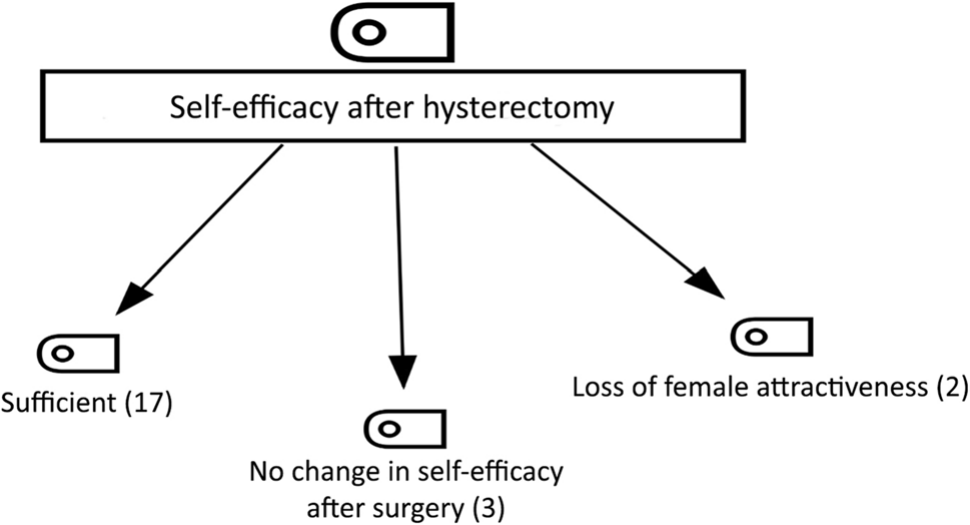
Hierarchical Code Sub-Code Model of Self-Efficacy After Hysterectomy

The hierarchical code-subcode model of ‘self-efficacy after hysterectomy’, another theme of the research, is shown in Fig. 5. The codes belonging to the theme (n = 22) are ‘sufficient’, ‘no change in self-efficacy after surgery’, and ‘loss of female attractiveness’.

The most intensely expressed code of the ‘self-efficacy after hysterectomy’ theme was ‘sufficient’ (n = 17). The statements of the participants coded P4 and P17 are as follows:

P4 (age: 62; gravida: 5): *I have always been a good mother and wife.*

P17 (age: 53; gravida: 4): *I educated and raised my children; I got them married off. Now I am looking after my grandchildren. Throughout my life, I thought about them more than myself and tried to be enough.*

In the code ‘no change in self-efficacy after surgery’ (n = 3), the statements of the participants coded P12 and P5 are as follows:

P12 (age: 50; gravida: 8): *I think that femininity will not change with the removal of the uterus, that I am still a woman.*

P5 (age: 50; gravida: 4): *Since I know my competence does not consist of my sex organs, I feel as competent after the operation as I felt before.*

In the ‘loss of female attractiveness’ code (n = 2), the statements of the participants coded P20 and P21 are as follows:

P20 (age: 45; gravida: 3): *I said that my hormonal balance was activated immediately because my breasts were immediately stretched, my stomach was flattened, and I had a very old body. I told my husband that we are not very old, but now I will clean the house, cook well, converse and listen, and continue the relationship.*

P21 (age: 50; gravida: 3): *I am scared. I have reached menopause but feel incomplete because my female organ was removed. Everything related to femininity was taken away from me due to this surgery.*

## Discussion and Conclusions

Hysterectomy is an operation that affects women emotionally and psychologically. In most cultures, the uterus is a symbol of femininity and fertility. Therefore, women’s perceptions of the operation vary from culture to culture (Hsieh et al. 2021; Skorupska et al. 2021). When the women participating in this study were asked about the meaning of femininity, the themes of ‘being a mother’, ‘being a good wife, a good mother’, ‘difficulty’, and ‘omnipotent’ came to the fore (Fig. 4). In previous studies, the concept of femininity has often been associated with sexuality, housewife, wife, and mother in terms of sexual status, capacity, and potential in both biological and social dimensions (Harford and Redmond 2021; Turco 2022). In this study, it was found that the ‘uterus was expressed as fertility’, ‘femininity’, ‘motherhood’, ‘menstruation’, ‘sexuality’, ‘a dirty and smelly organ’, ‘any organ’ (Fig. 3). In the literature, it has been reported that the uterus is seen as a fertility organ, sexual organ, secretory organ, regulator of body functions, and source of energy-health, youth, beauty, attractiveness, and power. Many women believe that the best days of their lives will end with a hysterectomy and define the operation as a loss of youth, femininity, and health (Choi et al. 2020; Hassan et al. 2022; Li et al. 2023). Although there are differences from culture to culture, similar aspects of the meanings women universally attribute to the uterus are noteworthy.

In our study results, the codes that women expressed positively after hysterectomy were ‘solution-oriented approach to the disease’, ‘getting rid of a sick body’, ‘no menstruation’, and ‘end of fertility’. In the ‘solution-oriented approach to the disease’ code, women (n = 12) stated that they saw this operation as a solution to their existing diseases and also expressed that they were pleased to get rid of the diseases when they had this operation (Fig. 1). Li et al. (2023) found that uterus-related diseases effectively influenced the preference for hysterectomy. Women perceive hysterectomy operations as an intervention to help them return and continue their daily lives (Goudarzi et al. 2021; Li et al. 2023). In a qualitative study, participants stated, *“I got out of that sick status and became a healthy person who could easily do any task”* (Goudarzi et al. 2021). In line with these findings, it can be considered that women perceive hysterectomy operation positively because it affects their quality of life.

In another positive code, ‘getting rid of a sick body’, participants (n = 9) expressed that they were glad that they no longer experienced the problems they had experienced before the operation (Fig. 1). A study observed that some women developed positive coping strategies after hysterectomy surgery (Li et al. 2023). Women mentioned the positive results of hysterectomy surgery by comparing the quality of life before and after surgery rather than the adverse effects of hysterectomy surgery (Li et al. 2023). Goudarzi et al. (2021) reported that women said about hysterectomy in their study, *“My uterus was a diseased organ in pain and bleeding. I was also worried about diseases such as infection, cervical cancer, and myoma. Now that I have had a hysterectomy, I am happy. I am free of a sick organ”*. It was determined that the study’s results were consistent with the literature.

In the literature, it has been reported that there are women who perceive the end of fertility positively and negatively (Choi et al. 2020; Hassan et al. 2022; Li et al. 2023). Similarly, in our study, the end of fertility was perceived positively by some participants, while some perceived it negatively (Fig. 1). One study revealed that the end of fertility after a hysterectomy did not cause worry and anxiety in women with children (Li et al. 2023). Another study found that women who had a hysterectomy were positive about it. They were relieved of problems such as the fear of unwanted pregnancy and the responsibility of using contraception regularly (Shirinkam et al. 2018). A study reported that age also influenced this attitude (Goudarzi et al. 2021). The study included the following statements, *“I am still young. I want to have another baby. When I have these thoughts, I get very angry. I get so upset. I struggle with myself and think about it. I am still young, but I cannot give birth to a baby. I think about it a lot. These thoughts disappoint me.”* (Goudarzi et al. 2021). It can be assumed that women’s attitudes to fertility loss parallel their desire to have children.

In this study, the ‘negative aspects’ code of the theme of ‘hysterectomy experiences’ was divided into sub-codes as ‘lack of organ’, ‘physical condition after hysterectomy’, ‘menopause’, ‘changing body image’, ‘changing self-perception’, ‘disruption in sexual pattern’ (Fig. 1). In the ‘lack of organ’ sub-code, the participants (n = 3) expressed sadness because they had lost their uterus and felt empty. Previous studies have reported that the uterus plays an important role in the perception of femininity and that removing it makes women feel defective and lose their womanhood (Goudarzi et al. 2021). Abadi et al. (2018) indicated that the physical and psychological changes that occur after losing female organs, such as the uterus and ovaries, cause women to have negative feelings about themselves. Women no longer see themselves as perfect women after the loss of their genitals. In another study, women expressed discomfort and suffering due to the absence of an organ after a hysterectomy (Silva and Vargens 2016). Merighi et al. (2012), who addressed the issue from a different aspect, found that the absence of the uterus was perceived as a threat of losing the partner and a defect that ended the marriage. In an interview conducted after the hysterectomy, the following statements were made by women, *“Removing the uterus affects women’s morale. I think a woman whose uterus is removed loses her femininity. I told my husband that I had become like a male person. Well, a feminine organ has been removed”*, and *“I used to think that the sense of femininity was related to the uterus, but after two years, I find that it is not so”* (Goudarzi et al. 2021). When the results of the research were analysed, it was found that the meaning that women attributed to the uterus was an important factor in their post-operative perceptions.

In this study, in the sub-code ‘physical condition after hysterectomy’, participants (n = 3) stated that they had complaints such as pain, gas pains, and nausea. One study reported that the most common complaints of women after a hysterectomy were pain, insomnia, eating disorders, and immobility; these conditions negatively affected women’s daily lives (Goudarzi et al. 2021). In the same study, participants reported, *“In fact, I was in a lot of pain after the removal of the uterus. I was like a sick person for a long time. It took me a long time to get my physical health back. Now, I cannot say that I consider myself a very healthy person. I feel that my health has decreased.”* (Goudarzi et al. 2021). As with all surgical procedures, gynaecological surgery is associated with physical complaints, but surgery that affects a woman’s hormonal balance, such as a hysterectomy, is associated with somatic complaints.

It is reported that women experience this process more severely than natural menopause, especially after a hysterectomy, which causes surgical menopause (Pinar et al. 2012; Erdogan et al. 2020). In the code ‘menopause’ (n = 3), another sub-code in the negative code, it was noted that women complained of menopausal symptoms after hysterectomy and were bothered by symptoms such as hot flushes and sweating. It has been shown in the literature that the physical and psychological changes caused by menopausal symptoms after a hysterectomy lead to a decline in marital relationships and negative self-esteem (Campbell et al. 2017). From a psychodynamic point of view, this situation can be linked to the loss of productivity and femininity during menopause (Choi et al. 2020; Hassan et al. 2022; Li et al. 2023).^.^ Further, studies have reported that hysterectomy has a negative impact on women’s body image and self-esteem (Pinar et al. 2012; Erdogan et al. 2020). The physical and psychological changes that occur after the loss of the uterus cause women to feel negative about themselves (Abadi et al. 2018). In this study, in the codes’ change in body image’ (n = 2) and ‘change in self-perception (n = 1), which are subcodes of the negative code of the theme ‘hysterectomy experience’, women expressed their negative feelings after hysterectomy, such as sagging breasts and not liking their body as much as before. Studies have shown that most women have negative feelings about perceived changes in their bodies (Pilli et al. 2020; Goudarzi et al. 2021). It is possible that hysterectomy may have a negative effect on women’s self-perception and body image because it is the cause of surgical menopause.

After hysterectomy, sexual dysfunction may occur, such as vaginal dryness due to estrogen deficiency, decreased sexual desire and interest, dyspareunia, decreased orgasm and sexual satisfaction (Kok et al. 2020). In this study, the number of women who thought that they would be affected (n = 6) and those who thought that they would not be affected (n = 6) were the same in the code ‘the effect of hysterectomy on sexual life’. When the sub-codes were analysed, it was found that the women who thought that their sexual life would be affected were due to pain in sexual intercourse, feeling of inadequacy, reluctance in sexual intercourse, fear in sexual intercourse, and lack of information about sexual life (Fig. 2). There have been reports in the literature that some women’s sexual life and marital relationships may be adversely affected after surgical menopause (Silva and Vargens 2016; Pillay and Manyonda 2022). Based on the findings, it is recommended that women be counseled about sexuality before having a hysterectomy.

In the sub-code of ‘thinking that sexuality will not be affected’ (n = 6), concepts such as having sufficient knowledge, not liking sexuality, and not wanting sexuality due to advanced age came to the fore. In our study, we found that receiving reliable information from the right source and spousal support positively influenced this process. The current study found that women received information about post-operative sexuality from friends, neighbours and other health professionals. Askew and Zam (2013) found that some women believed that a hysterectomy would have no effect on their sex life and that the operation revealed strengths in their relationship with their spouse. In a qualitative study by Kok et al. (2020), a woman stated, *“My husband supported me a lot in this process. We talked to him before the surgery, and he said that my health was more important than anything else and that he wanted me to have surgery. There will be no change in our sexual life after the surgery. I am very lucky in this regard, my husband cares a lot about me”*. Careful counselling and support from the spouse help women to make the right decision about the operation and to avoid psychological concerns as a strong support for accepting physical changes (Li et al. 2023). It can be thought that the situation of women’s sexuality being affected is parallel to the reliability of spousal support and information sources.

As a result of the findings from this study, it is recommended that health professionals counsel women undergoing hysterectomies. Women should be encouraged to express their feelings by allowing them to express their emotions, identifying women’s negative emotions, and teaching effective coping mechanisms.

## Data Availability

The data that support the findings of this study are available on request from the corresponding author.
